# Making brain monitoring routine: why processed EEG monitoring should be standard practice

**DOI:** 10.1007/s10877-026-01453-9

**Published:** 2026-06-05

**Authors:** Joana Berger-Estilita, Anthony Ray Absalom, Adrian W. Gelb

**Affiliations:** 1 Institute for Medical Education, University of Bern, Mittelstrasse 43, +41 31 684 62 01, Bern, 3012 Switzerland; 2 Salem Spital, Hirslanden Medical Group, Bern, Switzerland; 3 Centre for Health Technology and Services Research, Faculty of Medicine, RISE-Health, University of Porto, Porto, Portugal; 4 Department of Anesthesiology, University Medical Center Groningen, University of Groningen, Groningen, The Netherlands; 5 Department of Anesthesia and Perioperative Care, University of California San Francisco, San Francisco, CA USA

**Keywords:** Electroencephalography, Anesthesia, general, Monitoring, intraoperative, Patient safety, Postoperative delirium

## Abstract

Despite major technological advances and a growing evidence base, perioperative electroencephalographic (EEG) monitoring remains underused, even though general anaesthesia is fundamentally a brain-targeted intervention. Processed EEG (pEEG) captures clinically relevant brain physiology and provides actionable information to guide anaesthetic dosing, yet its adoption has been hindered by expectations of outcome certainty that exceed those applied to other standard monitors. Much of the controversy reflects misaligned endpoints—such as movement or binary awareness detection—rather than the domains in which EEG plausibly adds value, including avoidance of excessively deep anaesthesia and optimisation of drug titration. While large trials have produced heterogeneous results and have not demonstrated uniform benefit across broad surgical populations, some analyses have explored the possibility that EEG-guided anaesthesia may be more relevant in selected high-risk groups, particularly older adults vulnerable to postoperative neurocognitive disorders. Continued failure to integrate routine brain monitoring into standard anaesthetic practice may therefore pose a greater patient safety risk than adopting it in the setting of imperfect yet biologically coherent evidence.

## Introduction

Electroencephalographic (EEG) monitoring, and specifically processed EEG (pEEG), has long been anticipated to change titration of hypnotic drugs from a practice reliant on indirect indicators to one grounded in data. However, as of 2026, despite significant technological advancements and an expanding clinical evidence base, pEEG monitoring is not yet standard practice in many operating rooms [1, 2]. This ongoing implementation gap extends beyond academic debate. Given that general anesthesia is inherently a brain-targeted intervention, the lack of routine brain monitoring may constitute a significant oversight in perioperative safety and quality.

The primary purpose of monitoring is to furnish clinicians with timely information that cannot be directly observed, thereby supporting improved decision-making and patient outcomes. A pragmatic framework outlines three criteria for intraoperative monitoring [3]. First, the monitor must capture essential physiological parameters. Second, the measured parameter should be modifiable in a manner that plausibly enhances safety and outcomes. Third, the use of the monitor, along with appropriate clinical responses, should lead to improved patient-centered outcomes.

pEEG monitoring clearly satisfies the first two criteria. It assesses clinically significant electro-physiological parameters of the primary organ affected by general anesthesia and provides actionable data to guide drug titration, prevent excessive dosing, and identify abnormal brain states. Controversy arises with the third criterion because large outcome studies have yielded heterogeneous and often negative findings.

This shortcoming is not unique to pEEG. What is unique to pEEG is that it has been held to more rigorous standards than those applied to many established monitoring modalities. Many foundational technologies in anesthetic practice were adopted before the formalization and widespread adoption of evidence-based medicine (EBM). Pulse oximetry, capnography, and non-invasive blood pressure monitoring became standard due to their compelling physiological rationale and intuitive face validity, even without large randomized clinical trials demonstrating reductions in major clinical endpoints [4]. In the case of blood pressure monitoring coupled with active blood pressure management, the recent publication of a large trial showing that proactive avoidance of hypotension was futile in terms of clinical benefit has not dampened the enthusiasm with which anesthetists continue to measure blood pressure and take steps to avoid or limit hypotension [5]. 

Although end-tidal vapor monitoring and processed EEG emerged in a broadly similar period, they have been subject to different levels of scrutiny. This difference likely reflects not only historical context but also the nature of the variables being measured. End-tidal anesthetic gas monitoring aligns with the neurophysiological effects of the drugs, whereas pEEG attempts to characterize brain state—an inherently more complex and variable construct. In addition, pEEG was at times positioned as a “detector of consciousness”, raising expectations that exceed those applied to capnometry.

While this scrutiny is appropriate, it has also led to an excessive expectation that pEEG monitoring must demonstrate universal and consistent outcome benefits across all settings before it is recognized as a standard of care, and therefore, this article examines why that implementation gap persists and argues that continued failure to adopt pEEG monitoring as a standard of care may be undermining the safety and quality of perioperative care.

## The evidence: what pEEG can—and cannot—do

### Construct validity and the “depth of anaesthesia” problem

A central challenge for intraoperative pEEG monitoring is *construct validity*—the extent to which a measurement truly captures the underlying construct it is intended to represent. In practice, pEEG is often expected to reflect “depth of anaesthesia,” or more precisely, the absence of consciousness. However, consciousness remains a difficult construct to define, understand, and detect reliably, especially in clinical practice [6, 7]. It is therefore unsurprising that early validation studies focused on whether pEEG indices change systematically with progressive sedation. These investigations demonstrated statistical associations between increasing sedation scores and pEEG values [8, 9], but also revealed considerable overlap in index values across clinical states—raising concerns about limited sensitivity and specificity for any single threshold intended to map a specific depth category.

Current standards for perioperative monitoring reflect a significant evidentiary double standard between “basic” monitors and pEEG. While mandatory devices like pulse oximeters are cleared by the FDA and ISO based on technical accuracy—specifically, a root-mean-square error of 3% or less [10–12]—rather than on demonstrated reductions in mortality or major morbidity, pEEG is frequently dismissed for lacking universal “outcome certainty”. Historically, basic monitors such as capnography and non-invasive blood pressure (NIBP) were grandfathered into standard practice based on physiological face validity, bypassing the large-scale RCTs currently demanded for pEEG. Consequently, major guidelines from bodies like the WHO-WFSA [13] and the Association of Anaesthetists [14] categorize cardiovascular and respiratory monitors as “essential”. In contrast, pEEG is often relegated to an “optional” status, even though it monitors the primary target organ. This inconsistency is further highlighted by the critique that pEEG fails to predict movement, while NIBP is rarely criticized for its inability to predict consciousness, despite being used as a proxy for it.

Expecting a pEEG index to deterministically classify the depth of sedation and anesthesia across all drugs, patients, and clinical contexts is an unrealistic standard that is not applied to other physiological monitors.”

### Awareness under anesthesia: early promise, imperfect tests, and what trials really compared

Firstly, many studies assessed correlations between intraoperative bispectral index (BIS) values and the probability of explicit recall during the postoperative period [8]. This occurred during an era of increased attention to accidental awareness during intended general anesthesia (AAGA)—a complication that represents failure to achieve a primary goal of anesthesia (unconsciousness), with substantial consequences for patients and society [15–17]. Predictably, vendors promoted pEEG monitoring as a means of preventing AAGA, and multiple studies followed, most using postoperative detection of explicit recall as the primary endpoint. While a well-conducted randomized trial in patients at high risk of AAGA demonstrated benefit [18], subsequent trials have not consistently replicated this finding [19, 20]. Importantly, these differences are not explained by patient population alone, as several of these studies also enrolled high-risk patients, but rather by variations in study design—particularly the comparator strategy. For example, trials comparing pEEG-guided care with usual practice differ fundamentally from those comparing it with end-tidal anesthetic concentration–guided protocols, which themselves represent an active, protocolized strategy.

Second, several trials effectively compared *alarm strategies* rather than coherent *monitoring-informed clinical strategies* [19, 21]. In some designs, clinicians in the pEEG-guided group were broadly encouraged to reduce volatile anesthetic delivery based on pEEG information and clinical judgement, but were not provided standardized response algorithms; meanwhile, the comparator arm relied on end-tidal agent concentration alarms. In practice, that comparison is vulnerable to real-world feasibility issues: lower-limit end-tidal alarms are not routinely preset on anesthesia machines, many clinicians do not know how to configure them, and alarm use itself may be inconsistent to avoid alarm fatigue.

Thirdly, these trials effectively compared two imperfect surrogates for the brain effect: pEEG indices versus end-tidal anesthetic concentration targets. Both the minimal alveolar concentration (MAC) and pEEG thresholds have distributions rather than sharp cutoffs, as one would expect in the general population [22, 23]. Additionally, both MAC targets and pEEG-derived indices represent probabilistic surrogates of brain effect that vary across individuals and clinical contexts. As expected for physiological measures applied to heterogeneous patient populations, neither provides a deterministic threshold that guarantees a specific brain state. Under such conditions, the absence of a large difference between strategies is not necessarily evidence that EEG adds no value; it may simply reflect that the trial compared two “noisy” control strategies, neither of which was implemented as a tightly protocolized, brain-centered approach.

Fourthly, pEEG monitoring should not be reduced to the interpretation of a single numerical index, in the same way that use of the ECG for rate determination without considering wave morphology would represent an inadequate and inappropriate use of the monitoring modality. While processed indices may provide a convenient summary signal, meaningful clinical interpretation requires integrating multiple pEEG representations, including the raw waveform and time–frequency displays such as spectrograms or density spectral arrays (DSAs). Developing competency in recognizing these patterns is therefore essential for the safe and effective use of intraoperative pEEG [24]. 

Finally, controversies surrounding burst suppression further complicate interpretation: commercial devices commonly underestimate the degree of suppression; [25] Studies differ on whether they quantify cumulative suppression time or the number of suppression epochs; [26, 27] and the clinical meaning of suppression during induction may differ from that during maintenance [28]. 

### Movement as an outcome: limitations of cortical monitoring

A series of smaller studies used the isolated forearm technique (IFT) to assess responsiveness to command during intended anesthesia, with pEEG indices as secondary outcomes [29–32]. These studies often involved intentionally low anesthetic dosing and commonly reported that pEEG indices predicted command-following poorly. Several studies using the IFT have demonstrated that patients may follow commands during anesthesia, yet later report no recall of intraoperative events. How such episodes should be interpreted in terms of subjective experience and patient-centered outcomes remains an active area of investigation [32–34], as the IFT is not in routine use anywhere in the world.

Responsiveness detected using the IFT reflects a degree of preserved cortical processing and voluntary command-following during anesthesia [35]. This phenomenon should be distinguished from movement in response to noxious surgical stimulation, which is largely mediated through spinal reflex pathways and can occur even in the absence of cortical consciousness. For this reason, pEEG-derived indices, which primarily reflect cortical activity, are not expected to reliably predict motor responses to surgical stimulation.

Nonetheless, this mismatch between following commands and no recall highlights a recurring theme: a pEEG monitored should not be reduced to a binary awareness detector, and studies that use intraoperative responsiveness as an endpoint may be measuring a phenomenon that does not clearly map onto patient-centered harm.

Several early studies evaluated movement to noxious stimulation as an outcome and explored whether pEEG indices could predict it [36–38]. Larger studies later confirmed that pEEG indices—reflecting primarily cortical activity—are poor predictors of movement [39, 40]. This is unsurprising: it is well known that movement to pain can occur even with profound cortical suppression [41]. Indeed, nociceptive motor responses may be elicited in brain-dead patients [42]. Failure to predict movement is therefore not a failure of brain monitoring per se; it reflects that pEEG was asked to predict a physiology it does not directly measure.

The evidence base is more consistent for secondary outcomes that align with what pEEG plausibly enables: drug titration to brain effect. A substantial body of work indicates that pEEG-guided care reduces anesthetic dosing and is associated with modest improvements in recovery and discharge times, as well as lower postoperative nausea and vomiting [43, 44]. These benefits may not be dramatic in every setting, but they are clinically meaningful and mechanistically coherent, and savings in drug costs may help offset the monitor’s cost.

### Avoiding excessively deep anesthesia: mixed overall results, persistent signals in vulnerable cohorts

Excessively deep anesthesia—often defined in studies as a state occurring when there are very low pEEG indices or burst suppression—has been associated with adverse outcomes, including mortality and postoperative neurocognitive dysfunction [26, 45–47]. More recent research has therefore focused on whether pEEG-guided strategies that reduce the depth of anesthesia improve cognitive and other patient-centered outcomes. Overall, the evidence remains heterogeneous, with large trials showing reductions in pEEG suppression but inconsistent effects on postoperative delirium and other patient-centered outcomes [48–50]. Some secondary and exploratory analyses have examined whether pEEG-guided strategies might have differential effects in specific patient subgroups. For example, a post hoc analysis of the ENGAGES trial explored outcomes in older patients and suggested a potential association between EEG-guided care and reduced postoperative delirium in that subgroup [48]. In addition, a planned sub-study of the Balanced RCT reported that avoiding deep anesthesia reduced the incidence of postoperative delirium [26]. 

pEEG monitoring may help clinicians recognize and potentially avoid excessively deep cortical suppression during anesthesia, which has been associated with postoperative delirium in some observational studies [26]. These findings are consistent with evidence syntheses, including a Cochrane review and a more recent meta-analysis, which show that, in elderly or otherwise high-risk cohorts, pEEG monitoring is associated with a reduced incidence of postoperative delirium [51, 52]. 

All in all, the literature seems to suggest a familiar pattern in perioperative medicine: pEEG monitoring may not yield perfectly uniform benefit across all populations and endpoints, but it may offer meaningful gains where risk is concentrated—precisely the patients in whom anesthetists most need physiological guidance and in whom neurocognitive disorders carry the greatest individual and societal cost.

## Why the hesitation persists (Table 1)

**Table 1:** Barriers to Implementation of pEEG Monitoring

Barrier	Why it matters
Lack of standardized training	EEG interpretation requires specific cognitive skills that are not uniformly taught or assessed in anaesthesia training. It is also not routinely taught in medical school (in contrast to ECG), meaning many clinicians lack foundational familiarity with signal interpretation. Although processed indices exist (e.g., BIS, PSI), raw EEG and DSA interpretation remain mandatory but unfamiliar to many.
Perceived complexity and time constraints	In a high-pressure operating theatre environment, tools are adopted only if they provide immediate, visible benefit. EEG is often perceived as “too complex” or “unnecessary,” largely reflecting educational gaps rather than technological limitations.
Variability of devices and indices	Heterogeneity of EEG monitoring devices, proprietary indices, and lack of consensus thresholds for intervention contribute to confusion and reduced clinician trust. The absence of unified guidelines leads to inconsistent implementation across settings.
Cost considerations	While many modern anaesthesia workstations can integrate processed EEG modules, disposable sensor costs remain a perceived barrier—especially in low-resource settings. Sensor costs must be weighed against potential savings from anesthetic agents and reductions in downstream costs associated with adverse outcomes (delayed PACU discharge, delirium, prolonged hospitalisation, cognitive decline).
Cultural resistance	Anaesthesia has traditionally relied on indirect proxies (heart rate, blood pressure, MAC values). Introducing EEG-based titration challenges established mental models and routines, creating resistance to adoption despite potential value for individualized anesthetic care.
Implementing behavior change takes time	Changing human behavior can be frustratingly slow and requires committed, patient leadership. Engagement of implementation or human factors experts may facilitate adoption.

Given that the brain is the primary target of general anesthesia and that pEEG indices correlate with anesthetic effects on neuronal activity, why has routine EEG monitoring not become a standard?

Skepticism among many anesthetists is partly historical. The early adoption of pEEG was complicated by a sociological “clash of professional identity”. In the late 1990s, the aggressive commercial marketing of the first pEEG monitors—often implying that clinical signs alone were insufficient for safe practice—was perceived by many in the academic community as an undermining of traditional clinical expertise. This fostered a persistent legacy of skepticism. However, in 2026, the argument for pEEG has shifted from a commercial “need for help” to a scientific requirement for “organ-specific data”. Just as the use of an arterial line is not an admission of a clinician’s inability to feel a pulse, pEEG monitoring should be viewed not as a replacement for clinical judgment, but as the essential tool for monitoring the primary target organ of anesthesia.

In addition, while concerns about sensitivity and specificity are legitimate [53–55], they are often applied inconsistently. Anesthesia is filled with “fuzzy logic,” probabilistic reasoning, and context-dependent interpretation. A heart rate of 90 beats per minute may be too high, too low, or appropriate depending on circumstances; yet clinicians do not reject heart rate monitoring because it lacks universal thresholds. It is also illogical to demand that a processed EEG index deliver a single, absolute range that guarantees a fixed state of consciousness across all drugs, populations, and clinical contexts.

Another barrier is that the evidence base is not uniformly positive across traditional headline outcomes—movement, awareness, and neurocognitive dysfunction— leading professional societies to recommend selective use of pEEG monitoring in specific clinical contexts rather than universal implementation [56]. Most guidelines, at best, recommend pEEG in selected settings, such as during total intravenous anesthesia with neuromuscular blockade [14, 57]. This cautious stance reinforces the perception that EEG is an optional add-on rather than a foundational monitor. The dichotomy is striking. Cardiovascular and respiratory monitoring is mandated universally, despite these being effects of anesthetic drugs on several organs and systems, whereas monitoring of the target organ, the brain, remains optional [13, 58, 59]. 

Anesthetic practice and drug development have focused on brain pharmacology without directly coupling it to brain physiology. A frequently cited argument is that clinical signs—blood pressure, heart rate, tearing, sweating—are sufficient indicators of anesthetic state. The evidence does not support this view [9]. Traditional signs are poor predictors of brain state and are strongly influenced by spinal responses influenced by analgesics, autonomic tone, surgical stimulation, and vasoactive drugs [60]. Yet the “clinical signs are enough” narrative persists, perhaps because it is familiar and cognitively simple.

Finally, pEEG is challenged by the complexity of what it represents. Consciousness remains scientifically challenging to define and measure reliably, particularly in the context of anesthesia, where behavioral responses are suppressed. pEEG patterns vary with age, drugs, comorbidities, and baseline brain vulnerability. In comparison, blood pressure is a concept taught early, reinforced constantly, and interpreted within a naively uncomplicated framework. Processed EEG indices also have recognized limitations. For example, electromyographic activity can influence index values, and reductions in EMG activity following neuromuscular blockade may lower index values even without changes in cortical brain state. Experimental work by Schuller and colleagues [61] demonstrated that BIS values may decrease substantially after neuromuscular blockade alone, highlighting the susceptibility of index-based monitoring to non-cortical influences. These limitations reinforce the need to interpret pEEG indexes cautiously and to integrate multiple pEEG representations, including spectral edge frequency and DSA, rather than relying solely on a single numerical index. So, a monitor that requires training and pattern recognition—rather than threshold thinking—faces predictable resistance unless standards evolve to drive education and adoption [62]. 

## The patient safety argument: why waiting may be the greater risk

Monitoring is, at its core, a safety intervention. It provides additional information, while the clinician decides whether and how to respond. The decision to adopt a monitor should therefore be guided by a balanced assessment of plausible benefits and harms.

Processed EEG monitoring is non-invasive, low-risk, and rarely associated with clinically meaningful adverse effects. The plausible benefits are also substantial: improved titration, avoidance of excessive anesthetic dosing, identification of vulnerable brain states, and possible reductions in complications such as delirium and awareness.

Importantly, postoperative delirium, postoperative cognitive decline, and awareness are not rare curiosities. They are common, under-recognized, distressing, and costly—to patients, families, caregivers, and healthcare systems [63, 64]. As the surgical population ages and procedures become more complex, the case for routine brain monitoring strengthens rather than weakens. Persisting with indirect proxy measures while leaving the target organ largely unmonitored risks accepting potentially preventable harm as “normal variability.”

This perspective aligns with the emerging concept of perioperative “brain health,” which emphasizes the preservation of cognitive function across the surgical pathway and highlights the importance of strategies aimed at minimizing neurophysiological insult [65]. If personalized, brain-sensitive anesthesia is truly a goal of modern practice, it cannot succeed without routine measurement of brain state.

## The role of professional societies in guiding implementation

National and global anesthesia societies and hospital credentialling bodies should take a leadership role in normalizing perioperative pEEG monitoring. Current reluctance to include pEEG within basic monitoring standards often reflects a desire for definitive, uniform evidence. Yet requiring definitive RCT-level outcome evidence before integrating a monitoring technology into clinical practice may sometimes delay evaluation of tools that provide direct physiological information. In the case of pEEG monitoring, the proposed rationale primarily concerns its ability to provide real-time information about cortical activity and to support the titration of hypnotic drugs during anesthesia. However, the extent to which such monitoring translates into improved patient-centered outcomes remains uncertain and continues to be actively investigated.

To achieve the same “unquestioned” status as pulse oximetry or capnography, pEEG must transcend its current perception as a complex ‘add-on’. This requires a shift from ‘threshold-based’ monitoring (relying on a proprietary index) to ‘pattern-based’ monitoring (interpreting the raw EEG physiology) [62]. Effective implementation of pEEG monitoring depends on clinician education and competency in EEG interpretation. Recent work has highlighted structured educational initiatives aimed at improving EEG literacy among anesthesiologists [66]. Importantly, several contemporary reviews emphasize that meaningful use of EEG extends beyond reliance on processed indices and requires engagement with raw waveforms and time–frequency representations [67, 68]. Real change will occur when anesthesiologists view the EEG waveform as an essential vital sign of the target organ, rather than a black-box recommendation.

By incorporating brain monitoring into minimum monitoring recommendations—paired with clear competency expectations—professional societies would accelerate uptake, drive investment in training, and support innovation toward interpretable, standardized, clinically meaningful pEEG outputs. Above all, it would send a clear message: protecting the brain is not optional, but central to safe modern anesthesia.

As a final remark, while this work has focused primarily on processed EEG indices, it is important to recognize that raw EEG represents the most direct measure of brain activity during anesthesia. The perception that raw EEG is difficult to interpret is not unique, but reflects a broader issue of training and familiarity. Many routinely used monitoring modalities, including electrocardiography, require education and pattern recognition to yield clinically meaningful information. As brain monitoring becomes more integrated into routine practice, greater emphasis on training in raw EEG interpretation may further enhance clinicians’ ability to deliver truly brain-informed anesthesia.

## Conclusion

The persistent underuse of perioperative pEEG reflects a gap between what is technically feasible and what is routinely implemented at the bedside. Closing this gap is not simply about adopting a new technology; it is about strengthening the safety, quality, and dignity of anaesthetic care. Even if the evidence remains heterogeneous, there is a clear rationale for moving forward with a structured, pragmatic implementation.

## Data Availability

No datasets were generated or analysed during the current study.

## References

[1] Kane AD, Soar J, Armstrong RA, Kursumovic E, Davies MT, Oglesby FC, et al. Patient characteristics, anaesthetic workload and techniques in the UK: an analysis from the 7th National Audit Project (NAP7) activity survey. Anaesthesia. 2023;78(6):701–11. 10.1111/anae.15989. 36857758

[2] Pandit JJ, Andrade J, Bogod DG, Hitchman JM, Jonker WR, Lucas N, et al. 5th National Audit Project (NAP5) on accidental awareness during general anaesthesia: summary of main findings and risk factors†‡. BJA: Br J Anaesth. 2014;113(4):549–59. 10.1093/bja/aeu313. 25204697

[3] Meng L, Gelb AW. Oximetría cerebral: tres preguntas esenciales. Revista Colombiana de Anestesiología. 2015;43:52–6.

[4] Wollner E, Nourian MM, Booth W, Conover S, Law T, Lilaonitkul M, et al. Impact of capnography on patient safety in high- and low-income settings: a scoping review. Br J Anaesth. 2020;125(1):e88–103. 10.1016/j.bja.2020.04.057. Epub 20200427. 32416994

[5] Kant M, van Klei WA, Hollmann MW, de Klerk ES, Otterspoor LC, Besselink MG, et al. Proactive vs Reactive Treatment of Hypotension During Surgery: The PRETREAT Randomized Clinical Trial. JAMA. 2025;334(21):1905–14. 10.1001/jama.2025.18007. 41076587 PMC12516513

[6] Melloni L, Mudrik L, Pitts M, Koch C. Making the hard problem of consciousness easier. Science. 2021;372(6545):911–2. 10.1126/science.abj3259. Epub 2021/05/29. 34045342

[7] Coppola P. A review of the sufficient conditions for consciousness. Neurosci Biobehav Rev. 2025;177:106333. Epub 20250813. 10.1016/j.neubiorev.2025.106333. PubMed PMID: 40816661. 40816661

[8] Glass PS, Bloom M, Kearse L, Rosow C, Sebel P, Manberg P. Bispectral analysis measures sedation and memory effects of propofol, midazolam, isoflurane, and alfentanil in healthy volunteers. Anesthesiology. 1997;86(4):836–47. 9105228 10.1097/00000542-199704000-00014

[9] Struys MM, Jensen EW, Smith W, Smith NT, Rampil I, Dumortier FJ, et al. Performance of the ARX-derived auditory evoked potential index as an indicator of anesthetic depth: a comparison with bispectral index and hemodynamic measures during propofol administration. Anesthesiology. 2002;96(4):803–16. 11964586 10.1097/00000542-200204000-00006

[10] Hess DR. Pulse Oximetry: 2023 Year in Review. Respir Care. 2024;69(8):1033–41. Epub 20240724. doi: 10.4187/respcare.12023. PubMed PMID: 38806220; PubMed Central PMCID: PMCPMC11298236. 38806220 10.4187/respcare.12023 PMC11298236

[11] Sjoding MW, Iwashyna TJ, Valley TS. Change the Framework for Pulse Oximeter Regulation to Ensure Clinicians Can Give Patients the Oxygen They Need. Am J Respir Crit Care Med. 2023;207(6):661–4. 10.1164/rccm.202209-1773ED PubMed PMID: 36260769; PubMed Central PMCID: PMCPMC10037469. 36260769 PMC10037469

[12] Verkruysse W, Bartula M, Bresch E, Rocque M, Meftah M, Kirenko I. Calibration of Contactless Pulse Oximetry. Anesth Analg. 2017;124(1):136–45. 10.1213/ane.0000000000001381 PubMed PMID: 27258081; PubMed Central PMCID: PMCPMC5145250. 27258081 PMC5145250

[13] Gelb AW, Morriss WW, Johnson W, Merry AF, World Health Organization-World Federation of Societies of Anaesthesiologists (WHO-WFSA). International Standards for a Safe Practice of Anesthesia. Can J Anaesth. 2018;65(6):698–708. 10.1007/s12630-018-1111-5. Epub 20180507. 29736769

[14] Klein AA, Meek T, Allcock E, Cook TM, Mincher N, Morris C, et al. Recommendations for standards of monitoring during anaesthesia and recovery 2021: Guideline from the Association of Anaesthetists. Anaesthesia. 2021;76(9):1212–23. 10.1111/anae.15501. Epub 20210520. 34013531

[15] Lennmarken C, Bildfors K, Enlund G, Samuelsson P, Sandin R. Victims of awareness. Acta Anaesthesiol Scand. 2002;46(3):229 – 31. 10.1034/j.1399-6576.2002.t01-1-460301.x. PubMed PMID: 11939910. 11939910

[16] Sandin RH, Enlund G, Samuelsson P, Lennmarken C. Awareness during anaesthesia: a prospective case study. Lancet. 2000;355(9205):707–11. 10.1016/S0140-6736(99)11010-9 PubMed PMID: 10703802. 10703802

[17] Sandin R. Outcome after awareness with explicit recall. Acta Anaesthesiol Belg. 2006;57(4):429–32. PubMed PMID: 17236646. 17236646

[18] Myles PS, Leslie K, McNeil J, Forbes A, Chan MT. Bispectral index monitoring to prevent awareness during anaesthesia: the B-Aware randomised controlled trial. Lancet. 2004;363(9423):1757–63. 10.1016/S0140-6736(04)16300-9 PubMed PMID: 15172773. 15172773

[19] Avidan MS, Jacobsohn E, Glick D, Burnside BA, Zhang L, Villafranca A, et al. Prevention of intraoperative awareness in a high-risk surgical population. N Engl J Med. 2011;365(7):591–600. doi: 10.1056/NEJMoa1100403. PubMed PMID: 21848460. 21848460 10.1056/NEJMoa1100403

[20] Mashour GA, Shanks A, Tremper KK, Kheterpal S, Turner CR, Ramachandran SK, et al. Prevention of intraoperative awareness with explicit recall in an unselected surgical population: a randomized comparative effectiveness trial. Anesthesiology. 2012;117(4):717–25. 10.1097/ALN.0b013e31826904a6. Epub 2012/09/20. 22990178 PMC3447261

[21] Avidan MS, Zhang L, Burnside BA, Finkel KJ, Searleman AC, Selvidge JA, et al. Anesthesia awareness and the bispectral index. N Engl J Med. 2008;358(11):1097–108. doi: 10.1056/NEJMoa0707361. PubMed PMID: 18337600. 18337600 10.1056/NEJMoa0707361

[22] Dwyer R, Bennett HL, Eger EI 2nd, Heilbron D. Effects of isoflurane and nitrous oxide in subanesthetic concentrations on memory and responsiveness in volunteers. Anesthesiology. 1992;77(5):888–98. 10.1097/00000542-199211000-00009 PubMed PMID: 1443742. 1443742

[23] Aranake A, Mashour GA, Avidan MS. Minimum alveolar concentration: ongoing relevance and clinical utility. Anaesthesia. 2013;68(5):512–22. 10.1111/anae.12168. Epub 2013/02/19. 23414556

[24] Nimmo AF, Absalom AR, Bagshaw O, Biswas A, Cook TM, Costello A, et al. Guidelines for the safe practice of total intravenous anaesthesia (TIVA). Anaesthesia. 2019;74(2):211–24. 10.1111/anae.14428. 30378102

[25] Muhlhofer WG, Zak R, Kamal T, Rizvi B, Sands LP, Yuan M, et al. Burst-suppression ratio underestimates absolute duration of electroencephalogram suppression compared with visual analysis of intraoperative electroencephalogram. Br J Anaesth. 2017;118(5):755–61. 10.1093/bja/aex054 PubMed PMID: 28486575; PubMed Central PMCID: PMCPMC6224027. 28486575 PMC6224027

[26] Evered LA, Chan MTV, Han R, Chu MHM, Cheng BP, Scott DA, et al. Anaesthetic depth and delirium after major surgery: a randomised clinical trial. Br J Anaesth. 2021;127(5):704–12. 10.1016/j.bja.2021.07.021. Epub 20210828. 34465469 PMC8579421

[27] Shivdat S, Zhan T, De Palma A, Zheng WL, Krishnamurthy P, Paneerselvam E, et al. Early Burst Suppression Similarity Association with Structural Brain Injury Severity on MRI After Cardiac Arrest. Neurocrit Care. 2025;42(1):175–84. 10.1007/s12028-024-02047-6. Epub 20240724. 39043984 PMC11757804

[28] Obert DP, Sepúlveda PO, Adriazola V, Zurita F, Brouse J, Schneider G, et al. Overcoming age: Slow anesthesia induction may prevent geriatric patients from developing burst suppression and help developing intraoperative EEG signatures of a younger brain. J Clin Anesth. 2024;99:111672. 10.1016/j.jclinane.2024.111672. 39481245

[29] Russell IF. The ability of bispectral index to detect intra-operative wakefulness during total intravenous anaesthesia compared with the isolated forearm technique. Anaesthesia. 2013;68(5):502–11. 10.1111/anae.12177. Epub 20130322. 23521699

[30] Russell IF. The ability of bispectral index to detect intra-operative wakefulness during isoflurane/air anaesthesia, compared with the isolated forearm technique. Anaesthesia. 2013;68(10):1010–20. 10.1111/anae.12357 PubMed PMID: 24047289. 24047289

[31] Russell IF, Sanders RD. Monitoring consciousness under anaesthesia: the 21st century isolated forearm technique. Br J Anaesth. 2016;116(6):738–40. 10.1093/bja/aew112 PubMed PMID: 27199305. 27199305

[32] Sanders RD, Gaskell A, Raz A, Winders J, Stevanovic A, Rossaint R, et al. Incidence of Connected Consciousness after Tracheal Intubation: A Prospective, International, Multicenter Cohort Study of the Isolated Forearm Technique. Anesthesiology. 2017;126(2):214–22. 10.1097. Epub 2016/12/17. 27984262 10.1097/ALN.0000000000001479

[33] Pandit JJ, Russell IF, Wang M. Interpretations of responses using the isolated forearm technique in general anaesthesia: a debate. Br J Anaesth. 2015;115 Suppl 1:i32-i45. 10.1093/bja/aev106. PubMed PMID: 26174299. 26174299

[34] Russell IF. Midazolam-alfentanil: an anaesthetic? An investigation using the isolated forearm technique. Br J Anaesth. 1993;70(1):42–6. 10.1093/bja/70.1.42 PubMed PMID: 8431332. 8431332

[35] Lennertz R, Pryor KO, Raz A, Parker M, Bonhomme V, Schuller P, et al. Connected consciousness after tracheal intubation in young adults: an international multicentre cohort study. Br J Anaesth. 2023;130(2):e217–24. 10.1016/j.bja.2022.04.010. Epub 20220523. 35618535 PMC10375493

[36] Kearse LA Jr., Manberg P, Chamoun N, DeBros F, Zaslavsky A. Bispectral analysis of the electroencephalogram correlates with patient movement to skin incision during propofol/nitrous oxide anesthesia. Anesthesiology. 1994;81(6):1365–70. 7992904 10.1097/00000542-199412000-00010

[37] Vernon JM, Lang E, Sebel PS, Manberg P. Prediction of movement using bispectral electroencephalographic analysis during propofol/alfentanil or isoflurane/alfentanil anesthesia. Anesth Analg. 1995;80(4):780–5. 7893035 10.1097/00000539-199504000-00023

[38] Sebel PS, Bowles SM, Saini V, Chamoun N. EEG bispectrum predicts movement during thiopental/isoflurane anesthesia. J Clin Monit. 1995;11(2):83–91. PubMed PMID: 7760092. 7760092 10.1007/BF01617729

[39] Sebel PS, Lang E, Rampil IJ, White PF, Cork R, Jopling M, et al. A multicenter study of bispectral electroencephalogram analysis for monitoring anesthetic effect. Anesth Analg. 1997;84(4):891–9. 10.1097/00000539-199704000-00035 PubMed PMID: 9085977. 9085977

[40] Doi M, Gajraj RJ, Mantzaridis H, Kenny GN. Prediction of movement at laryngeal mask airway insertion: comparison of auditory evoked potential index, bispectral index, spectral edge frequency and median frequency. Br J Anaesth. 1999;82(2):203–7. 10.1093/bja/82.2.203 PubMed PMID: 10364994. 10364994

[41] Sonner JM, Antognini JF, Dutton RC, Flood P, Gray AT, Harris RA, et al. Inhaled anesthetics and immobility: mechanisms, mysteries, and minimum alveolar anesthetic concentration. Anesth Analg. 2003;97(3):718–40. PubMed PMID: 12933393. 12933393 10.1213/01.ANE.0000081063.76651.33

[42] Saposnik G, Basile VS, Young GB. Movements in brain death: a systematic review. Can J Neurol Sci. 2009;36(2):154 – 60. 10.1017/s031716710000651x. PubMed PMID: 19378707. 19378707

[43] Laferriere-Langlois P, Morisson L, Jeffries S, Duclos C, Espitalier F, Richebe P. Anesth Analg. 2024;138(2):295–307. Epub 20240112. doi: 10.1213/ANE.0000000000006860. PubMed PMID: 38215709. Depth of Anesthesia and Nociception Monitoring: Current State and Vision For 2050. 10.1213/ANE.0000000000006860 38215709

[44] Punjasawadwong Y, Chau-In W, Laopaiboon M, Punjasawadwong S, Pin-On P. Processed electroencephalogram and evoked potential techniques for amelioration of postoperative delirium and cognitive dysfunction following non-cardiac and non-neurosurgical procedures in adults. Cochrane Database Syst Rev. 2018;5(5):Cd011283. 10.1002/14651858.CD011283.pub2. Epub 20180515. 29761891 PMC6494561

[45] Monk TG, Saini V, Weldon BC, Sigl JC. Anesthetic management and one-year mortality after noncardiac surgery. Anesth Analg. 2005;100(1):4–10. E. PubMed PMID: 15616043. 15616043 10.1213/01.ANE.0000147519.82841.5E

[46] Sessler DI, Sigl JC, Kelley SD, Chamoun NG, Manberg PJ, Saager L, et al. Hospital stay and mortality are increased in patients having a triple low of low blood pressure, low bispectral index, and low minimum alveolar concentration of volatile anesthesia. Anesthesiology. 2012;116(6):1195–203. 10.1097/ALN.0b013e31825683dc PubMed PMID: 22546967. 22546967

[47] Fritz BA, Kalarickal PL, Maybrier HR, Muench MR, Dearth D, Chen Y, et al. Intraoperative Electroencephalogram Suppression Predicts Postoperative Delirium. Anesth Analg. 2016;122(1):234–42. PubMed PMID: WOS:000366710000004. 26418126 10.1213/ANE.0000000000000989 PMC4684753

[48] Wildes TS, Mickle AM, Ben Abdallah A, Maybrier HR, Oberhaus J, Budelier TP, et al. Effect of Electroencephalography-Guided Anesthetic Administration on Postoperative Delirium Among Older Adults Undergoing Major Surgery: The ENGAGES Randomized Clinical Trial. JAMA. 2019;321(5):473–83. PubMed PMID: 30721296; PubMed Central PMCID: PMCPMC6439616. 30721296 10.1001/jama.2018.22005 PMC6439616

[49] Deschamps A, Ben Abdallah A, Jacobsohn E, Saha T, Djaiani G, El-Gabalawy R, et al. Electroencephalography-Guided Anesthesia and Delirium in Older Adults After Cardiac Surgery: The ENGAGES-Canada Randomized Clinical Trial. JAMA. 2024;332(2):112–23. PubMed PMID: 38857019; PubMed Central PMCID: PMCPMC11165413. 38857019 10.1001/jama.2024.8144 PMC11165413

[50] Short TG, Campbell D, Frampton C, Chan MTV, Myles PS, Corcoran TB, et al. Anaesthetic depth and complications after major surgery: an international, randomised controlled trial. Lancet. 2019;394(10212):1907–14. 10.1016/S0140-6736(19)32315-3. Epub 20191020. 31645286

[51] Lewis SR, Pritchard MW, Fawcett LJ, Punjasawadwong Y. Bispectral index for improving intraoperative awareness and early postoperative recovery in adults. Cochrane Database Syst Rev. 2019;9(9):CD003843. 10.1002/14651858.CD003843.pub4. Epub 20190926. 31557307 PMC6763215

[52] Meng L, Zhao X, Sun Y, Cheng S, Bao L, Fang K, et al. Characteristics associated with effectiveness in postoperative delirium research: a systematic review of randomised controlled trials with meta-regression and meta-analysis. Br J Anaesth. 2024;133(3):565–83. 10.1016/j.bja.2024.05.033. Epub 20240704. 38969535

[53] Kalkman CJ, Drummond JC. Monitors of depth of anesthesia, quo vadis? Anesthesiology. 2002;96(4):784–7. 10.1097/00000542-200204000-00003 PubMed PMID: 11964583. 11964583

[54] Drummond JC. Monitoring depth of anesthesia: with emphasis on the application of the bispectral index and the middle latency auditory evoked response to the prevention of recall. Anesthesiology. 2000;93(3):876–82. 10.1097/00000542-200009000-00039 PubMed PMID: 10969323. 10969323

[55] Gaskell A, Sanders RD, Sleigh J. Using EEG markers to titrate anaesthesia. Br J Anaesth. 2018;121(1):327–9. 10.1016/j.bja.2018.04.003. 29935588

[56] Practice advisory for. intraoperative awareness and brain function monitoring: a report by the american society of anesthesiologists task force on intraoperative awareness. Anesthesiology. 2006;104(4):847–64. 10.1097/00000542-200604000-00031 PubMed PMID: 16571982. 16571982

[57] Nimmo AF, Absalom AR, Bagshaw O, Biswas A, Cook TM, Costello A, et al. Guidelines for the safe practice of total intravenous anaesthesia (TIVA): Joint Guidelines from the Association of Anaesthetists and the Society for Intravenous Anaesthesia. Anaesthesia. 2019;74(2):211–24. 10.1111/anae.14428. Epub 2018/11/01. 30378102

[58] Aldecoa C, Bettelli G, Bilotta F, Sanders RD, Aceto P, Audisio R, et al. Update of the European Society of Anaesthesiology and Intensive Care Medicine evidence-based and consensus-based guideline on postoperative delirium in adult patients. Eur J Anaesthesiol. 2024;41(2):81–108. 10.1097/eja.0000000000001876. Epub 20230830. 37599617 PMC10763721

[59] Chan MTV, Hedrick TL, Egan TD, García PS, Koch S, Purdon PL, et al. American Society for Enhanced Recovery and Perioperative Quality Initiative Joint Consensus Statement on the Role of Neuromonitoring in Perioperative Outcomes: Electroencephalography. Anesth Analg. 2020;130(5):1278–91. 10.1213/ane.0000000000004502 PubMed PMID: 31764163. 31764163

[60] Flaishon R, Windsor A, Sigl J, Sebel PS. Recovery of consciousness after thiopental or propofol. Bispectral index and isolated forearm technique. Anesthesiology. 1997;86(3):613–9. 9066327 10.1097/00000542-199703000-00013

[61] Schuller PJ, Newell S, Strickland PA, Barry JJ. Response of bispectral index to neuromuscular block in awake volunteers. Br J Anaesth. 2015;115(Suppl 1):i95–103. 10.1093/bja/aev072 PubMed PMID: 26174308. 26174308

[62] Berger-Estilita J, Saxena S, Gisselbaek M. Advancing electroencephalography education in anesthesiology. Curr Opin Anaesthesiol. 2025;38(5):576–83. Epub 20250526. doi: 10.1097/aco.0000000000001521. PubMed PMID: 40421501; PubMed Central PMCID: PMCPMC12382741. 40421501 10.1097/ACO.0000000000001521 PMC12382741

[63] Gou RY, Hshieh TT, Marcantonio ER, Cooper Z, Jones RN, Travison TG, et al. One-Year Medicare Costs Associated With Delirium in Older Patients Undergoing Major Elective Surgery. JAMA Surg. 2021;156(5):430–42. 10.1001/jamasurg.2020.7260 PubMed PMID: 33625501; PubMed Central PMCID: PMCPMC7905699. 33625501 PMC7905699

[64] Weiss Y, Saxena S, Gisselbaek M, Berger-Estilita J, Matot I. The importance of the presence of chosen family in preventing peri-operative delirium. Eur J Anaesthesiol. 2025;42(6):488–91. 10.1097/eja.0000000000002169. Epub 20250507. 40308047

[65] Bonhomme V, Putensen C, Böttiger BW, Stevens MF, Marczin N, Arnal D, et al. Brain health: A concern for anaesthesiologists and intensivists. Eur J Anaesthesiol Intensive Care. 2024;3(6):e0063. 10.1097/ea9.0000000000000063 PubMed PMID: 02260475-202412000-00001. 39917635 PMC11798402

[66] Berger-Estilita J, Baron Shahaf D, Barreto Chang OL, Bonhomme V, Crisan I, Fernandez-Candil JL et al. Consensus document on electroencephalography education in anaesthesiology: defining learning outcomes: A modified four-round Delphi study. Eur J Anaesthesiol. 2026. Epub 20260219. doi: 10.1097/EJA.0000000000002346. PubMed PMID: 41709666. 10.1097/EJA.0000000000002346 PMC13566452 41709666

[67] Manohara N, Ferrari A, Greenblatt A, Berardino A, Peixoto C, Duarte F, et al. Electroencephalogram monitoring during anesthesia and critical care: a guide for the clinician. J Clin Monit Comput. 2025;39(2):315–48. 10.1007/s10877-024-01250-2. Epub 20241220. 39704777

[68] Guay CS, Agrawal U, Tseng B, Gallo S, Schreier DR, Brown EN. Clinical Electroencephalography for Anesthesiologists and Intensivists: Part 2-Physiologic Signatures and Active Management. Anesthesiology. 2025;143(6):1595–618. 10.1097/aln.0000000000005739. Epub 20251111. 41537509 PMC12594142

